# Pharmacological antagonism of kainate receptor rescues dysfunction and loss of dopamine neurons in a mouse model of human parkin-induced toxicity

**DOI:** 10.1038/s41419-020-03172-8

**Published:** 2020-11-10

**Authors:** Maria Regoni, Stefano Cattaneo, Daniela Mercatelli, Salvatore Novello, Alice Passoni, Renzo Bagnati, Enrico Davoli, Laura Croci, Gian Giacomo Consalez, Federica Albanese, Letizia Zanetti, Maria Passafaro, Giulia Maia Serratto, Alessio Di Fonzo, Flavia Valtorta, Andrea Ciammola, Stefano Taverna, Michele Morari, Jenny Sassone

**Affiliations:** 1 Division of Neuroscience, IRCCS San Raffaele Scientific Institute, Via Olgettina 58, 20132 Milan, Italy; 2 Vita-Salute San Raffaele University, Via Olgettina 58, 20132 Milan, Italy; 3 Department of Medical Sciences, Section of Pharmacology, University of Ferrara, Via Fossato di Mortara 17-19, 44121 Ferrara, Italy; 4 Department of Environmental Health Sciences, Istituto di Ricerche Farmacologiche Mario Negri IRCCS, Via Mario Negri 2, 20156 Milan, Italy; 5 CNR, Institute of Neuroscience, Milan, Via Luigi Vanvitelli 32, 20129 Milan, Italy; 6 Department of Neurology and Laboratory of Neuroscience, IRCCS Istituto Auxologico Italiano, Piazzale Brescia 20, 20149 Milan, Italy; 7 Foundation IRCCS Ca’ Granda Ospedale Maggiore Policlinico, Neurology Unit, Via Francesco Sforza 28, 20122 Milan, Italy; 8 Dino Ferrari Center, Department of Pathophysiology and Transplantation, University of Milan, Neuroscience Section, Via Francesco Sforza 28, 20122 Milan, Italy

**Keywords:** Parkinson's disease, Experimental models of disease

## Abstract

Mutations in the *PARK2* gene encoding the protein parkin cause autosomal recessive juvenile Parkinsonism (ARJP), a neurodegenerative disease characterized by dysfunction and death of dopamine (DA) neurons in the substantia nigra pars compacta (SNc). Since a neuroprotective therapy for ARJP does not exist, research efforts aimed at discovering targets for neuroprotection are critically needed. A previous study demonstrated that loss of parkin function or expression of parkin mutants associated with ARJP causes an accumulation of glutamate kainate receptors (KARs) in human brain tissues and an increase of KAR-mediated currents in neurons in vitro. Based on the hypothesis that such KAR hyperactivation may contribute to the death of nigral DA neurons, we investigated the effect of KAR antagonism on the DA neuron dysfunction and death that occur in the parkinQ311X mouse, a model of human parkin-induced toxicity. We found that early accumulation of KARs occurs in the DA neurons of the parkinQ311X mouse, and that chronic administration of the KAR antagonist UBP310 prevents DA neuron loss. This neuroprotective effect is associated with the rescue of the abnormal firing rate of nigral DA neurons and downregulation of GluK2, the key KAR subunit. This study provides novel evidence of a causal role of glutamate KARs in the DA neuron dysfunction and loss occurring in a mouse model of human parkin-induced toxicity. Our results support KAR as a potential target in the development of neuroprotective therapy for ARJP.

## Introduction

Mutations in the *PARK2* gene (OMIM 600116) cause the most common form of autosomal recessive juvenile Parkinsonism (ARJP), a neurodegenerative disease characterized by the loss of dopamine (DA) neurons in the substantia nigra pars compacta (SNc)^1^. The *PARK2* gene encodes parkin, a ubiquitin-ligase enzyme that catalyzes the transfer of ubiquitin to protein substrates, thus regulating their trafficking and turnover^2^. Many parkin substrates have been identified to date, indicating that parkin is a multifunctional protein involved in many intracellular processes, including the control of mitochondrial integrity and the regulation of apoptosis and transcription^3,4^. These studies notwithstanding how *PARK2* mutations lead to DA neuron death are still unclear. More importantly, a therapy that prevents or slows down the neurodegeneration in ARJP patients has not been developed yet.

Accruing evidence suggests that parkin plays an important role in the modulation of neurotransmission^5^. Parkin localizes at both the presynaptic terminals, where it interacts with synaptic vesicles^6^, and at the postsynaptic elements^7^, where it interacts with the synaptic scaffolding molecules PSD-95, CASK^7^, and PICK1^8^. A previous study reported that parkin interacts with and ubiquitinates the GluK2 subunit of the ionotropic glutamate kainate receptor (KAR), and that parkin regulates KAR currents in glutamatergic neurons^9^. Loss of parkin function was found to lead to GluK2 accumulation and increased KAR currents in glutamatergic neurons in vitro^9^. GluK2/KAR levels were increased in the brain tissues of parkin-knockout mice^10^ and ARJP patients bearing *PARK2* mutations^9^.

KARs are expressed in human DA neurons^11,12^ (http://www.braineac.org), but their precise functions in DA neuron physiology have not been established to date. Since the main effect of KAR activation in glutamatergic neurons is an increase of neuronal excitability^13,14^, it is conceivable that activation of postsynaptic KARs expressed on SNc DA neurons increases their excitability. Therefore, KAR accumulation in DA neurons of ARJP models and patients might contribute to DA neuron dysfunction and, ultimately, death.

Here, we investigated the potential role of KARs in the DA neuron dysfunction and loss that occur in the parkinQ311X mouse, a model of human parkin-induced toxicity.

## Materials and methods

### Animals

C57B1/6N ParkinQ311X mice, expressing the human parkin variant parkinQ311X^15^ selectively in DA neurons, were previously generated and characterized^16^ (The Jackson Laboratories, Bar Harbor, ME, USA). This mouse model has normal wild-type parkin alleles on both chromosomes in addition to the exogenous human parkinQ311X variant, which is ectopically expressed from a bacterial artificial chromosome and driven by the DA transporter (DAT) promoter. The phenotype of this mouse is due to a dominantly inherited toxic gain of function^16^. Gender-matched littermates were used as controls. All experiments were conducted with the aim of minimizing the number of sacrificed animals. Since *PARK2*-related ARJP is not sex-linked, and prevalence and disease symptoms do not differ between males and females, according to the principle of the 3Rs (replacement, reduction, and refinement), to minimize the number of animals used, both male and female animals (in equal ratio) were recruited in the study. Mice were maintained and bred at the animal house of Ospedale San Raffaele in compliance with institutional guidelines and international laws (EU Directive 2010/63/EU EEC Council Directive 86/609, OJL 358, 1, December 12, 1987, NIH Guide for the Care and Use of Laboratory Animals, U.S. National Research Council, 1996).

### Dissection of substantia nigra

Mice were anesthetized by intraperitoneal injection of a mixture of ketamine/xylazine (100 and 10 mg/kg, respectively, Sigma-Aldrich) and sacrificed by cervical dislocation. Brains were removed, placed in ice-cold PBS for 2–3 min, and then transferred to the platform of a tissue chopper (McIlwain^™^ Tissue Chopper). Tissue was positioned perpendicular to the chopper blade, and 300-μm-thick coronal slices were cut in less than 1 min. The brain slices were immediately transferred to PBS 1×. Substantia nigra was isolated under a dissecting microscope and immediately frozen in liquid nitrogen.

### Western blotting

Brain tissues were homogenized in ice-cold lysis buffer (50 mM Tris-HCl, pH 7.5, 150 mM NaCl, 1 mM EDTA, 1% NP-40, 0.5% sodium deoxycholate, and 0.1% sodium dodecyl sulfate (SDS)) added with Complete Protease Inhibitor Cocktail (Sigma). Lysates were incubated in ice for 30 min, sonicated in ice for 15 s with the ultrasonic processor UP100H (Hielscher) with a frequency of 30 kHz, and a power output of 80%, and centrifuged at 13,000 rpm at 4 °C for 20 min. Proteins in the supernatant were quantified by BCA assay (Thermo Scientific) according to the manufacturer’s instructions. Samples were diluted with 3× SDS loading buffer (188 mM Tris-HCl, pH 6.8, 6% SDS, 30% glycerol, and 0.3% bromophenol blue) and 1 M 1,4-dithiothreitol (the final concentration of DTT was 100 mM) and heated to 95 °C for 10 min. Protein extracts (20 μg) were separated on NuPAGE 4–12% Bis–Tris gels (Life Technologies) with NuPAGE MOPS SDS Running Buffer (Life Technologies) and the MagicMark XP Standard molecular weight marker (Life technologies). After electrophoresis, gels were transferred onto nitrocellulose membranes (GE Healthcare) for 2 h at 400-mA constant current at 4 °C in transfer buffer (25 mM Tris, 192 mM glycine, and 20% methanol). Post-transfer membranes were briefly incubated in Ponceau Solution (Sigma) to visualize proteins, rinsed in Tris-Buffered Saline containing 0.05% Tween-20 (TBS-T), blocked with 5% nonfat dried milk in TBS-T for 1 h at room temperature, and then incubated overnight at 4 °C with the following primary antibodies: anti-GluR6/7 (Origine cod. TA310550 1:1000), anti-GluA1 (Cell Signaling cod. 13185-D4N9V 1:2000), anti-GluA2 (NeuroMab clone L21/32 1:1000), anti-cleaved Caspase-3 (Cell Signaling cod. #9661 1:1000), anti-Fodrin (Cell Signaling cod. 2122 1:1000), anti-PARP (Cell Signaling cod.9542 1:1000), anti-TH (Millipore cod. MAB310 clone LNC1 1:1000), anti-GAPDH (Santa Cruz cod. sc-25778 1:1000), and anti-Tubulin β3 (Covance cod. MMS-435P: 1:10,000). Membranes were then washed 3 × 10 min in TBS-T, incubated with horseradish peroxidase (HRP)-conjugated secondary antibodies (GE Healthcare-Amersham Biosciences) for 1 h at room temperature, and then washed 3 × 10 min in TBS-T. The antigens were detected using an immunodetection kit (Novex ECL, Invitrogen) according to the manufacturer’s instructions. For visualization, we used the Chemidoc Touch Imaging System (BioRad). Band intensity was quantified by densitometry analysis using ImageJ software^17^. Briefly, the images were converted to an 8-bit format in order to perform uncalibrated optical density. After conversion, the background was subtracted through the rolling-ball radius method (default setting, ball radius value: 50.0 pixels). Each band was individually selected and circumscribed with the rectangular ROI tool selection. Data were acquired as arbitrary-integrated density values. The final relative expression level was normalized by the internal standard GAPDH.

### Immunofluorescence

Mice were deeply anesthetized by intraperitoneal injection of a mixture of ketamine/xylazine (100 and 10 mg/kg, respectively, Sigma-Aldrich) and transcardially perfused with ice-cold 0.9% NaCl solution, followed by 4% paraformaldehyde (PFA) in PBS (0.1 M, pH 7.4, Sigma-Aldrich, St. Louis, MO, USA). The brains were postfixed in 4% PFA overnight at 4 °C and then transferred to a 30% sucrose solution in PBS for cryoprotection and stored at −80 °C. Thirty-micrometer-thick sections of SNc were prepared using a freezing microtome, transferred to glass slides coated with polylysine, and dried at room temperature. Sections were then rehydrated, washed, and incubated in 10 mM sodium citrate buffer (pH 6) at 80 °C for 30 min (antigen retrieval), then kept for 20 min in ice. The slices were then treated with blocking solution, 5% normal goat serum (Sigma-Aldrich) added with 0.1% triton in PBS, and incubated for 48 h with 1:250 anti-GluR6/7 (04-921, Millipore, Burlington, MA, USA) and 1:500 anti-TH (Mab318, Millipore). After three washes with PBS, the tissues were incubated for 2 h at room temperature in the dark with secondary antibodies (1:300 goat anti-rabbit IgG Alexa 488 to reveal GluK2 and 1:300 goat anti-mouse IgG Alexa 546 to reveal TH, Invitrogen). After three washes in PBS, cell nuclei were counterstained with 4′,6-diamidino-2-phenylindole solution. Slides were mounted using the Dako fluorescence mounting medium (Dako). Images were acquired using a Leica TCS SP2 confocal microscope (Leica, Wetzlar, Germany) and analyzed using ImageJ software^17^. Both image acquisition and quantification were performed by investigators who were “blind” to the experimental condition.

### Stereological cell count in SNc

Stereological counting was performed according to published methods^18^. Mice were deeply anesthetized by intraperitoneal injection of a mixture of ketamine/xylazine (100 and 10 mg/kg, respectively, Sigma-Aldrich) and transcardially perfused with ice-cold 0.9% NaCl solution, followed by 4% PFA in PBS (0.1 M, pH 7.4, Sigma-Aldrich, St. Louis, MO, USA). The brains were postfixed in 4% PFA overnight at 4 °C and then transferred to a 30% sucrose solution in PBS for cryoprotection and stored at −80 °C. Fifty-micrometer-thick free-floating sections of SNc (AP from −3.16 to −3.52 from bregma^19^) were prepared using a freezing microtome. Sections were washed in PBS and incubated in 3% hydrogen peroxide/PBS for 10 min to eliminate endogenous peroxidase activity. After several washes, sections were incubated for 30 min at room temperature with blocking solution (PBS + BSA 1:50 + Triton X-100 0.3%) and then incubated overnight at 4 °C with TH antibody (ab112, 1:750 in BSA 1% PBST, Abcam, Cambridge, UK). Sections were then rinsed, incubated for 1 h with anti-rabbit HRP-conjugated secondary antibody (ab6721, 1:500 in BSA 1% PBST, Abcam), and revealed by a DAB substrate kit (ab64238, Abcam). Sections were mounted on slides coated with gelatine, dehydrated, and coverslipped. Stereological analysis was performed by counting TH + neurons (phenotypic marker) and cresyl violet-stained cells (structural marker) in the SNc. Neural cell counting was performed on 5 serial slices (magnified at 40×), which were cut 50-μm thick and 200-μm apart, by applying an unbiased stereological sampling method based on optical fractionator stereological probe^18,20,21^. A Leica DM600B motorized microscope equipped with a Stereo Investigator software (MBF Europe, Delft, The Netherlands) was used. The resulting number of neurons is expressed as a raw value deriving from software calculation that estimates the total number of neurons from the number of neurons within a Systematic Randomly Sampled set of unbiased virtual counting spaces covering the entire region of interest with a uniform distance between unbiased virtual counting in spaces in directions *X*, *Y*, and *Z*. Image acquisition and quantification were performed by investigators who were “blind” to the experimental condition.

### High-performance liquid chromatography-multiple reaction monitoring (HPLC-MRM) analysis of UBP310 concentration in brain tissues and in plasma

UBP310 (3-({3-[(2S)-2-Amino-2-carboxyethyl]-5-methyl-2,6-dioxo-3,6-dihydro-1(2H)-pyrimidinyl}methyl)-2-thiophenecarboxylic acid) was purchased from TOCRIS (Bristol, UK), dissolved in 100% dimethyl sulfoxide (DMSO) at a concentration of 10 mg/mL, and then diluted in 90% saline + 10% DMSO at 1 mg/mL. Mice were injected i.p. with UBP310 (20 mg/kg) or with an equivalent volume of vehicle. After 10–30–60–120–240–480–1440 min, animals were anesthetized by intraperitoneal injection of a mixture of ketamine/xylazine (100 and 10 mg/kg, respectively, Sigma-Aldrich) and sacrificed by cervical dislocation. Plasma and brain samples were collected and stored at −80 °C until analysis. Whole-brain samples were homogenized at the ratio of 100 mg tissue/1 mL of methanol/water 85:15 (v/v), containing 10 ng/mL of TRP-D5. Samples were placed for 20 min at −80 °C to allow the separation of fat materials. Homogenates were centrifuged for 15 min at 13,200 rpm at 4 °C, and 500 μL of the supernatants were dried under nitrogen and resuspended in 100 μL of chromatographic mobile phase for instrumental analysis. The analyses of UBP310 were performed using a 1200 Series HPLC system (Agilent Technologies, CA, USA) interfaced with an API 5500 triple-quadrupole mass spectrometer (Sciex, Thornhill, Ontario, Canada). The mass spectrometer was equipped with an electrospray ionization source and was operated in positive ion and multiple-reaction monitoring modes to measure the product ions formed in the collision cell from the molecular ions of the analytes. In the preliminary phase, standard solutions of UBP310 and tryptophan-D5 (TRP-D5), used as internal standard, were directly injected into the UBP spectrometer, to identify the best ion transitions for MRM acquisition. The identified transitions were m/z 354.1 –> 197.1 (quantification transition) and m/z 354.1 –> 308.1 (qualification transition) for UBP310; m/z 210.3 –> 150.1 (quantification transition) and m/z 210.3 -> 122.1 (qualification transition) for TRP-D5. The ion source settings were as follows: ion spray voltage, 5500 V; curtain gas, 28; collision gas, 7; source temperature, 320 °C; ion source gas 1 and gas 2, 50, and 40 psi, respectively. The HPLC separation of UBP310 and TRP-D5 was obtained with an Ascentis Express C18 column (150 × 2.1 mm; 2.7-μm particle size, Sigma-Aldrich, St. Louis, MO), using an elution mixture composed of solvent A (0.05% acetic acid in water) and solvent B (acetonitrile) at 30 °C. The elution gradient was from 1 to 99% of solvent B in 12 min; hold at 99% for 2 min and re-equilibrate for 5 min at 10% of solvent B. The injection volume was 5 μl and the flow rate was 180 μl/min.

### Chronic treatment with UBP310 or vehicle

UBP310 (3-({3-[(2S)-2-Amino-2-carboxyethyl]-5-methyl-2,6-dioxo-3,6-dihydro-1(2H)-pyrimidinyl}methyl)-2-thiophenecarboxylic acid) was purchased from TOCRIS (Bristol, UK), dissolved in 100% DMSO at a concentration of 10 mg/mL, and then diluted in 90% saline + 10% DMSO at 1 mg/mL. The mice were injected i.p. with UBP310 (20 mg/kg) or an equivalent volume of vehicle (90% saline + 10% DMSO) every day at the same hour (8–10 a.m.) for the duration of the treatment.

### Cell-attached and whole-cell patch-clamp recordings of DA neurons of the SNc in mouse brain slices

Twenty-five-day-old mice were anesthetized by intraperitoneal injection of a mixture of ketamine/xylazine (100 mg/kg and 10 mg/kg, respectively, Sigma-Aldrich) and perfused transcardially with ice-cold artificial cerebrospinal fluid (ACSF) containing (in mM) 125 NaCl, 3.5 KCl, 1.25 NaH_2_PO_4_, 2 CaCl_2_, 25 NaHCO_3_, 1 MgCl_2_, and 11 d-glucose, saturated with 95% O_2_ and 5% CO_2_ (pH 7.3). After decapitation, the brains were removed and 250-µm-thick coronal slices containing the SN were cut in a solution containing (in mM) 50 sucrose, 125 NaCl, 2.5 KCl, 1.25 NaH_2_PO_4_, 0.1 CaCl_2_, 25 NaHCO_3_, 6.2 MgCl_2_, and 2.5 d-glucose, saturated with 95% O_2_ and 5% CO_2_ (pH 7.3) at 4 °C using a VT1000S vibratome (Leica Microsystems, Wetzlar, Germany). Slices were kept in the cutting solution at 32 °C before being submerged in a recording chamber mounted on the stage of an upright BX51WI microscope (Olympus, Japan) equipped with differential interference contrast optics. Slices were continuously perfused with ACSF at a rate of 2–3 ml/min at 32 °C. DA neurons were recorded using glass pipettes filled with a solution containing the following (in mM): 10 NaCl, 124 KH_2_PO_4_, 10 HEPES, 0.5 EGTA, 2 MgCl_2_, 2 Na_2_-ATP, and 0.02 Na-GTP (pH 7.2, adjusted with KOH; tip resistance: 4–6 MΩ). Cells were recorded either in cell-attached configuration to detect spontaneous firing activity or whole-cell configuration to assess intrinsic membrane parameters. Slices were continuously perfused with normal ACSF as baseline activity of patched cells was recorded. Subsequently, ACSF perfusion was replaced with ACSF containing 5 μM UBP310 for 10 min, followed by ACSF containing 10 μM UBP310 for 10 additional minutes. Finally, perfusion was switched back to normal ACSF to wash out the drug for at least 15 min. After completing an experiment on any given cell, the slice was disposed to avoid potential drug interference with the following recordings. Traces were recorded using a MultiClamp 700B amplifier interfaced with a computer through a Digidata 1440A (Molecular Devices, Sunnyvale, CA, USA). Traces were sampled at a frequency of 10 kHz and low-pass filtered at 2 kHz. Data were acquired using pClamp10 software (Molecular Devices) and analyzed with Clampfit and GraphPad Prism (La Jolla, CA).

### Data presentation and statistical analysis

Data are presented as mean ± standard error of the mean (SEM). The normality test (Kolmogorov–Smirnov test) and equal variance test (Bartlett’s test) were applied. Two-tailed unpaired Student’s *t* test was used to compare two groups of data. One-way ANOVA followed by appropriate post hoc tests was used to compare more than two groups. The Friedman repeated-measure ANOVA on ranks was used on non-normally distributed datasets to analyze the effect of UBP310 on firing frequency.

## Results

### ParkinQ311X mice display early KAR accumulation associated with degeneration of SNc DA neurons

**Fig. 1:**
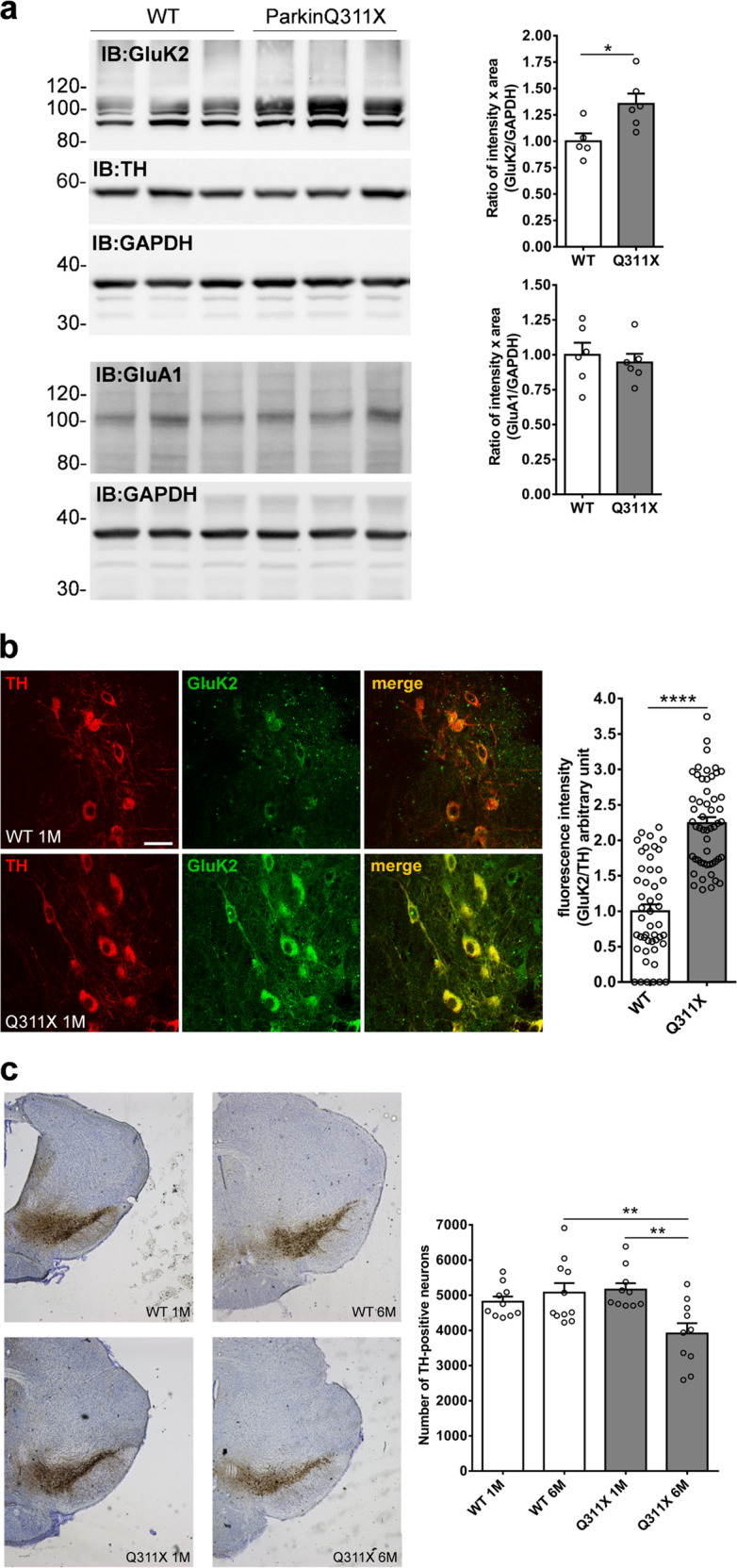
SNc DA neurons of parkinQ311X mice display early KAR accumulation and degeneration. **a** Representative Western blot showing the levels of GluK2 subunit of KAR and GluA1 subunit of AMPAR in lysates prepared from the substantia nigra of WT or parkinQ311X mice at 1 month of age. Membranes were also probed for TH as DA neuron marker. Samples were run in triplicate, with each lane loaded with lysate from an individual mouse. The histograms on the right show the means ± SEM from densitometer quantifications. For GluK2/KAR analysis, all the bands between 80 and 120 kDa were quantified. GluK2/KAR: WT 1.00 ± 0.07 *n* = 5 mice, parkinQ311X 1.35 ± 0.10 *n* = 6 mice, two-tailed unpaired Student *t* test *p = 0.012, *t* = 2.786, d*f* = 9; GluA1: WT 1.00 ± 0.09 *n* = 6 mice, parkinQ311X 0.94 ± 0.06 *n* = 6 mice, two-tailed unpaired Student *t* test *p* > 0.05. **b** Representative confocal images showing TH (red) and GluK2 (green) double immunofluorescence in the SNc of the WT and the parkinQ311X mice at 1 month of age. The image is representative of 12 sections derived from *n* = 3 WT mice and 12 sections from *n* = 3 parkinQ311X mice (GluK2 antibody Millipore 04-921). The bar is 30 µm. The histogram shows the means ± SEM of fluorescence intensity. Each data point on the graph represents one neuron. Twelve sections per genotype were examined, derived from *n* = 3 WT mice and *n* = 3 parkinQ311X mice. GluK2/TH: WT 1.00 ± 0.10 *n* = 48 neurons, parkinQ311X 2.24 ± 0.08 *n* = 53 neurons, two-tailed unpaired Student *t* test ****p = 3.87E–16, *t* = 9.749, d*f* = 99. Data from non-normalized GluK2 intensity: WT 1.00 ± 0.12 *n* = 48 neurons, parkinQ311X 3.22 ± 0.13 *n* = 53 neurons, two-tailed unpaired Student *t* test ****p = 5.03E−22, *t* = 12.47, d*f* = 99. **c** Representative images showing TH immunoperoxidase labeling in the SNc of the WT and the parkinQ311X mice at 1 and 6 months of age. The histograms just below the picture show DA neuron quantification performed by stereological count. Data are the means ± SEM of TH-positive neurons: WT 1 month (1 M) 4817 ± 146 *n* = 10 mice, parkinQ311X 1 M 5160 ± 182 *n* = 10 mice, WT 6 months (6 M) 5075 ± 270 *n* = 11 mice, and parkinQ311X 6 M 3913 ± 293 *n* = 10 mice, one-way ANOVA followed by Tukey’s test. *F* = 5.933, WT 6 M vs. parkinQ311X 6 M. **p = 0.0054, parkinQ311X 1 M vs. parkinQ311X 6 M, **p = 0.0033.

We previously found that *PARK2* mutations induce GluK2/KAR accumulation in rat neurons grown in vitro and in human brains^9^. To test whether *PARK2* mutations induce early accumulation of KAR specifically in nigral DA neurons, we analyzed GluK2/KAR levels in the isolated SNc of 1-month-old parkinQ311X mice and WT littermates (Supplementary Fig. S1a, b). Nigral GluK2/KAR levels were higher in the parkinQ311X mice than in WT mice. The GluA1 subunit levels of AMPAR were unchanged (Fig. 1a). Immunofluorescence for GluK2/KAR and TH in mesencephalic brain slices confirmed the GluK2/KAR accumulation in the DA neurons of the parkinQ311X mice (Fig. 1b).

An ~40% reduction of the number of DA neurons in the SNc of parkinQ311X mice at 16 months of age was previously reported^16^. We analyzed the number of nigral DA neurons in mice at younger ages. No differences were found between the parkinQ311X mice and their littermate controls at 1 month, and a 20% reduction in DA neurons was found at 6 months (Fig. 1c). These results confirm that the parkinQ311X mouse displays DA neuron loss^16^ and show that degeneration of DA neurons is not limited to aged animals. Importantly, GluK2/KAR accumulation precedes DA neuron degeneration in this model of human parkin-induced toxicity.

### UBP310 rescues the loss of SNc DA neurons in ParkinQ311X mice

We hypothesized that if GluK2/KAR accumulation contributes to DA neuron loss in parkinQ311X mice, then pharmacological antagonism of KAR might provide neuroprotection. UBP310 is a competitive orthosteric antagonist of KARs shown to antagonize KAR activity in vitro and in vivo^22,23^. Several lines of evidence show that UBP310 is a specific KAR antagonist^22–24^.

To quantify the bioavailability and distribution of peripherally administered UBP310 in the brain, WT mice were treated with a single intraperitoneal injection of UBP310 (20 mg/kg i.p.) and time-course analysis of UBP310 levels in the plasma and brain was performed. The UBP310 plasmatic peak occurred within 10 min from injection; UBP310 was quickly distributed in the brain, its concentration remained >100 ng of UBP310/g brain tissue for 1 h before slowly diminishing within 24 h (Supplementary Fig. S2). No abnormal behaviors or clinical signs were observed in the treated mice: all remained presumptively healthy without signs of debilitation, pain, or discomfort. Treatment did not change the levels of proteins associated with cell death (Supplementary Fig. S3a) or of neuronal and dopaminergic markers (Supplementary Fig. S3b), thus ruling out overt, acute UBP310 in vivo toxicity.

**Fig. 2:**
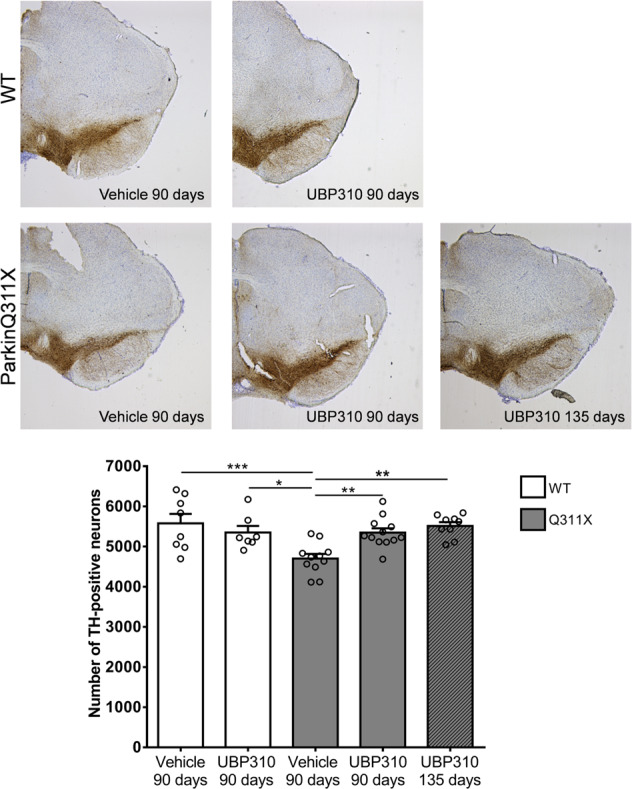
UBP310 rescues the loss of SNc DA neurons in parkinQ311X mice. Representative images showing TH immunoperoxidase labeling in the SNc of the WT and the parkinQ311X mice (6 months of age) treated with either vehicle or UBP310 (20 mg/kg i.p.) for 90 or 135 days. The graph below the picture shows DA neuron quantification performed by stereological count. Data are the means ± SEM of TH-positive neurons: WT treated with vehicle for 90 days 5578 ± 235 *n* = 8 mice, WT treated with UBP310 for 90 days 5351 ± 162 *n* = 7 mice, parkinQ311X treated with vehicle for 90 days 4701 ± 117 *n* = 11 mice, parkinQ311X treated with UBP310 for 90 days 5347 ± 107 *n* = 12 mice, parkinQ311X mice treated with UBP310 for 135 days 5514 ± 94 *n* = 9 mice, one-way ANOVA followed by Tukey’s test *F* = 6.681, WT vehicle 90 days vs. parkinQ311X vehicle 90 days ***p = 0.0007, WT UBP310 90 days vs. parkinQ311X vehicle 90 days *p = 0.0260, parkinQ311X vehicle 90 days vs. parkinQ311X UBP310 90 days **p = 0.0073, parkinQ311X vehicle 90 days vs. parkinQ311X UBP310 135 days **p = 0.0012.

Based on these results, we treated 3-month-old parkinQ311X mice and their age-matched WT littermates with UBP310 (20 mg/kg daily i.p.) or vehicle (10% DMSO in saline) for 90 days. Chronic treatment with either UBP310 or vehicle induced no mortality or weight changes (Supplementary Fig. S4). No signs of pain, distress, or toxicity were observed; animals fed, walked, and groomed normally. At the end of treatment, 6-month-old mice were sacrificed and brains analyzed for the number of SNc DA neurons. An ~20% reduction in DA neurons was observed in the parkinQ311X mice treated with vehicle, whereas no significant loss of DA neurons was found in the UBP310-treated parkinQ311X mice. To substantiate these data, a cohort of parkinQ311X mice was treated with UBP310 (20 mg/kg/day i.p) for 135 days, starting from 1.5 months of age. Again, no nigral DA neuron loss was observed, demonstrating that UBP310 is able to prevent SNc degeneration in parkinQ311X mice (Fig. 2).

### UBP310 normalizes the firing activity of DA neurons and downregulates GluK2 levels in ParkinQ311X mice

**Fig. 3:**
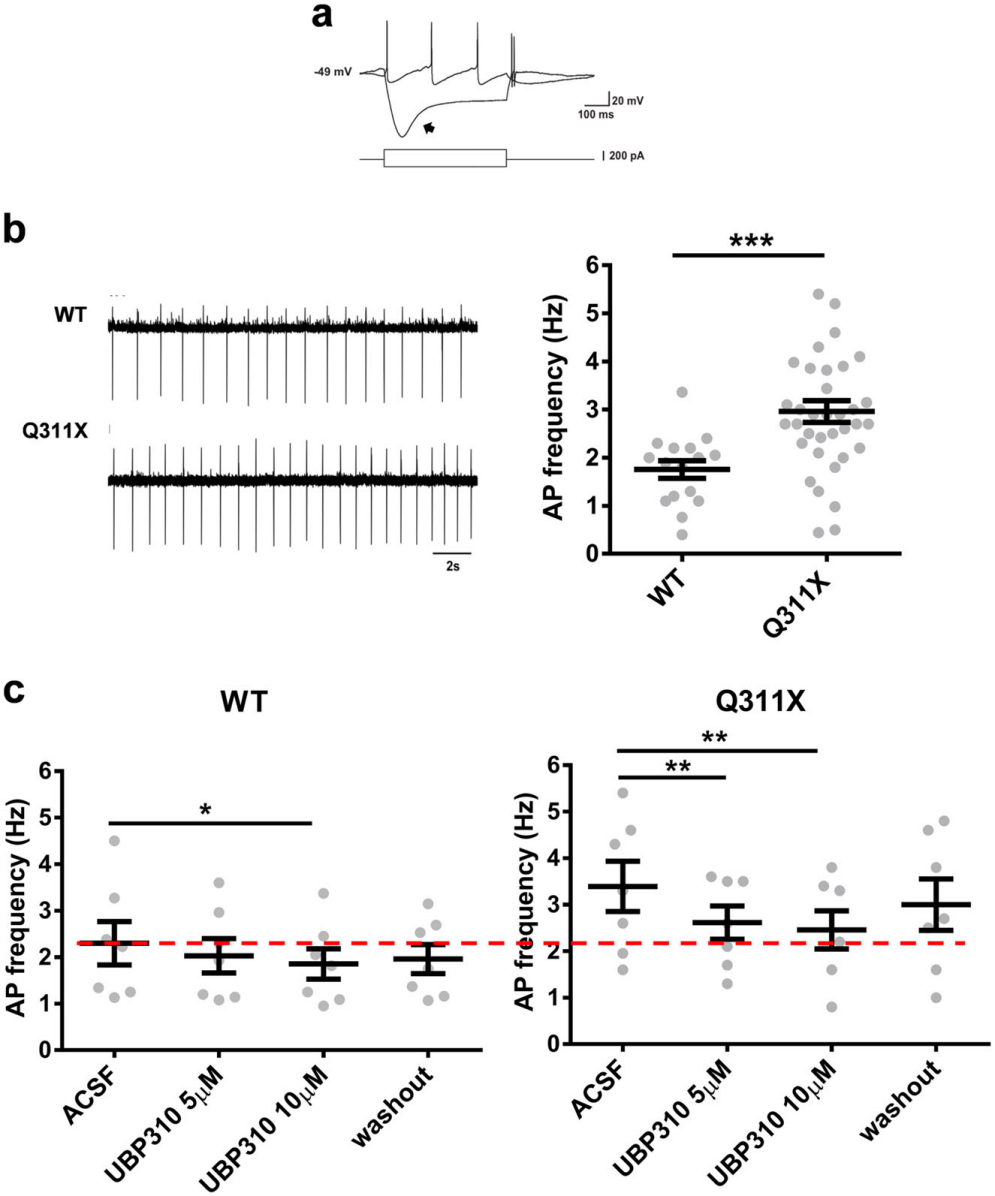
UBP310 normalizes the spontaneous firing activity of SNc DA neurons of the parkinQ311X mouse. **a** Examples of patch-clamp whole-cell recordings in current-clamp mode from an SNc DA neuron in an acute slice of ventral midbrain. The upper traces show membrane potential hyperpolarizing and depolarizing responses to intracellular injection of negative and positive current steps, respectively (±200 pA, 500 ms, lower traces). Note the relatively depolarized resting-membrane potential (−49 mV) and the prominent *I*_h_-dependent depolarizing “sag” (arrow) typical of DA neurons. **b** Cell-attached recordings of spontaneous action potential (AP) firing of SNc DA neurons from WT and parkinQ311X mice at 25 days of age. The dot plot on the right shows the quantification of spontaneous firing frequencies. The mean firing frequency of the SNc DA neurons was significantly higher in parkinQ311X mice compared to WT mice (mean ± SEM WT: 1.75 ± 0.18 Hz, parkinQ311X mice: 2.96 ± 0.23 Hz). Data were obtained from 6 WT mice (16 recorded neurons) and 8 parkinQ311X mice (36 recorded neurons). Unpaired Student *t* test with Welch correction, ***p = 0.0001, *t* = 4.148, d*f* = 47.75. **c** Summary dot plots showing spontaneous firing frequencies of SNc DA neurons in acute slices of ventral midbrain prepared from WT and parkinQ311X mice at 25 days of age upon extracellular perfusion with UBP310 (5 and 10 µM) and subsequent washout. Mean ± SEM, WT ACSF 2.3 ± 0.46 Hz, WT treated with UBP310 5 µM 2.03 ± 0.37 Hz, WT treated with UBP310 10 µM 1.85 ± 0.32 Hz, WT washout 1.96 ± 0.31 Hz; *p = 0.02, F = 7.83, Friedman repeated-measure ANOVA on ranks. Q311X ACSF 3.4 ± 0.5 Hz, Q311X treated with UBP310 5 µM 2.61 ± 0.36 Hz, Q311X treated with UBP310 10 µM 2.46 ± 0.41 Hz, Q311X washout 3.0 ± 0.55 Hz; one-way repeated-measure ANOVA Holm–Sidak test **p = 0.009, *F* = 11.87 (7 recorded neurons for each condition).

We investigated the mechanism by which UBP310 rescues DA neuron loss in parkinQ311X mice. The main effect of the glutamate-induced activation of KARs in hippocampal neurons is to increase neuronal excitability^13,14^. Conceivably, an increase in GluK2/KAR function at the glutamatergic postsynapse of SNc DA neurons might enhance DA neuron excitability, increase energy demand, and possibly result in cell death^25^. To test this hypothesis, we analyzed the electrophysiological properties of SNc DA neurons in the brain slices from parkinQ311X and WT mice. Intrinsic membrane properties did not differ between WT and parkinQ311X-DA neurons (Supplementary Fig. S5). However, the average spontaneous firing frequency was greater in parkinQ311X-DA neurons than WT DA neurons (Fig. 3a, b). To test whether this dysfunction was mediated by KAR currents, we recorded spontaneous firing activity upon acute UBP310 application to brain slices. In WT DA neurons, 5 µM UBP310 was ineffective, whereas 10 µM UBP310 slightly decreased the firing frequency. In parkinQ311X-DA neurons, both 5 and 10 µM UBP310 decreased the abnormal firing frequency to a level similar to WT control (Fig. 3c). These data demonstrate that pharmacological antagonism of KAR normalizes the abnormal firing frequency of *PARK2* neurons.

**Fig. 4:**
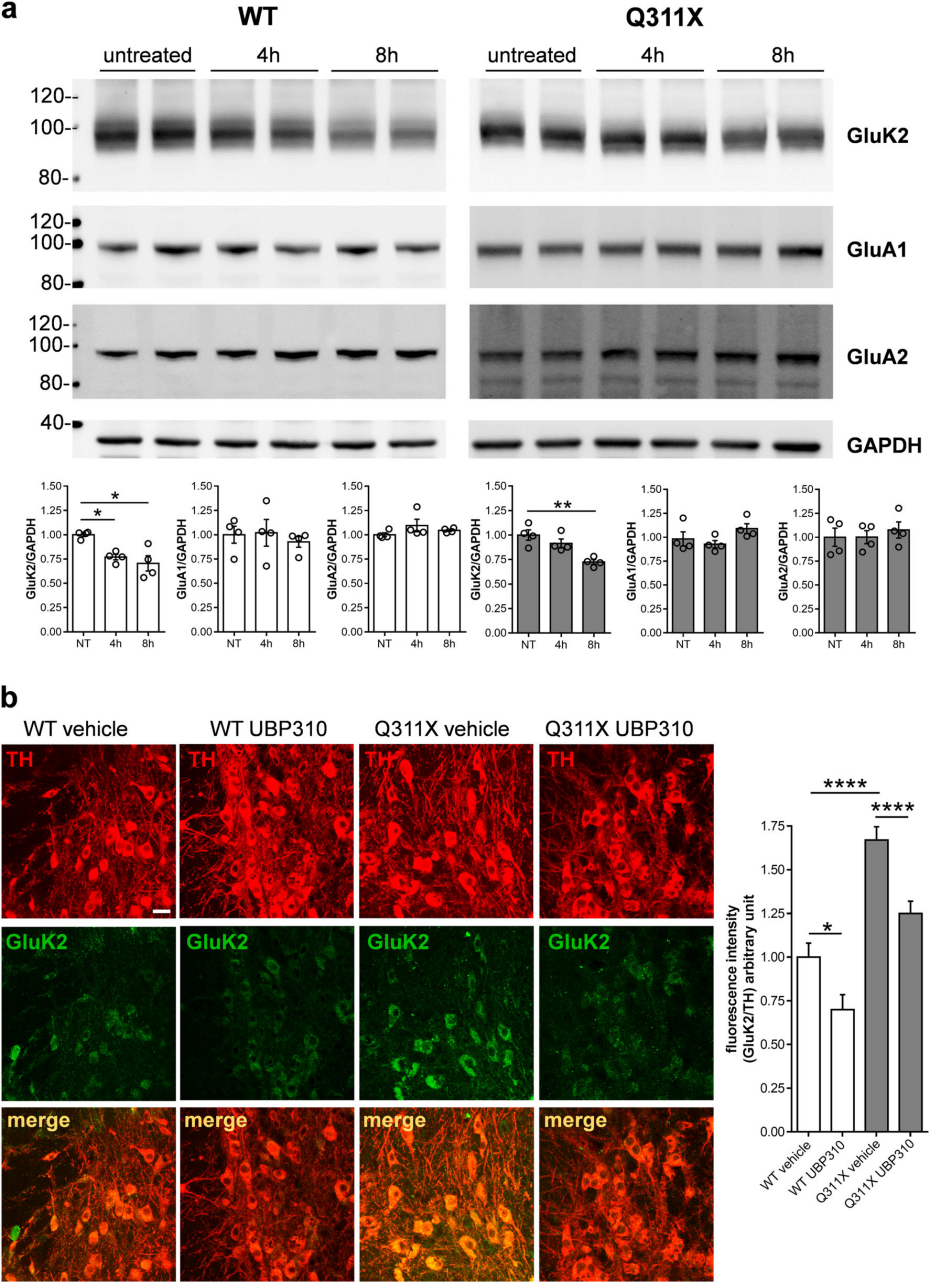
UBP310 decreases the level of the GluK2 subunit of KAR. **a** Representative Western blots showing GluK2 immunostaining from total brain lysates obtained from WT or parkinQ311X mice treated with 20 mg/kg UBP310 i.p. for either 4 or 8 h. The graphs show the means ± SEM from densitometer quantifications (WT—white bars, Q311X—gray bars). UBP310 treatment induced a decrease in GluK2 levels. WT mice, NT 1.00 ± 0.02, 4 h 0.77 ± 0.03, 8 h 0.71 ± 0.08, 4 h vs. NT *p = 0.047, 8 h vs. NT *p = 0.0011, *F* = 7.276, one-way ANOVA with Bonferroni’s test. ParkinQ311X mice, NT 1.00 ± 0.05, 4 h 0.92 ± 0.04, 8 h 0.73 ± 0.02, 8 h vs. NT **p = 0.0013, *F* = 13.36, one-way ANOVA with Bonferroni’s test. UBP310 did not change the levels of AMPA receptor subunits GluA1 and GluA2. **b** Representative confocal images showing TH (red) and GluK2 (green) double immunofluorescence in the SNc of WT or parkinQ311X mice treated with either vehicle or 20 mg/kg UBP310 i.p. for 8 h. Images were acquired using a Leica TCS SP2 confocal microscope, objective 40× (bar, 50 µm). The histograms show the means ± SEM from fluorescence-intensity quantifications. Twelve sections per genotype/treatment were examined derived from *n* = 3 WT mice treated with vehicle, *n* = 3 WT mice treated with UBP310, *n* = 3 parkinQ311X mice treated with vehicle, and *n* = 3 parkinQ311X mice treated with UBP310. GluK2/TH: WT vehicle 1.00 ± 0.08 *n* = 95 neurons, WT UBP310 0.7 ± 0.09 *n* = 91 neurons, parkinQ311X vehicle 1.67 ± 0.08 *n* = 102 neurons, parkinQ311X UBP310 1.25 ± 0.07 *n* = 100 neurons, WT vehicle vs. WT UBP310 *p = 0.039, Q311X vehicle vs. Q311X UBP310 *p* = 0.0006, WT vehicle vs. Q311X vehicle *p* = 0.00009, *F* = 27.95, one-way ANOVA followed by Tukey’s test.

Since pharmacological antagonism can induce receptor downregulation, we also tested whether KAR blockade by UBP310 was associated with GluK2/KAR downregulation. To do this, we analyzed GluK2/KAR levels in whole-brain lysates of WT and parkinQ311X mice acutely treated with 20 mg/kg UBP310 i.p., at 4 or 8 h after treatment. UBP310 induced a time-dependent decrease in GluK2 levels in both WT and parkinQ311X mice: WT mice showed a significant decrease in GluK2/KAR protein levels 4 and 8 h after treatment with UBP310; parkinQ311X mice showed a significant decrease in GluK2 protein levels 8 h after treatment with UBP310 (Fig. 4a). This effect was specific for KAR since UBP310 did not affect the levels of the AMPAR subunits GluA1 and GluA2. To confirm this result in DA neurons, we analyzed GluK2/KAR levels in mesencephalic brain slices by immunofluorescence. The experiments confirmed that UBP310 reduces GluK2/KAR levels in the DA neurons of WT and parkinQ311X mice (Fig. 4b). The data suggest that, by binding to KAR and blocking its opening, UBP310 can also induce degradation of the key subunit GluK2.

## Discussion

This study provides novel evidence of the role of KARs in the DA neuron dysfunction and loss occurring in the parkinQ311X mouse, a model of human parkin-induced toxicity.

KAR is a tetrameric receptor composed of various combinations of five subunits (named GluK1–5) expressed in many human brain regions, including SNc^12^ (http://www.braineac.org/). GluK2, which promotes the cell surface expression of the other subunits^26^, is the most abundant subunit in SNc^12^. Our result showing increased levels of GluK2/KARs in DA neurons of parkinQ311X mice is in agreement with previous studies demonstrating KAR accumulation in ARJP models and in human brain tissues from ARJP patients^9,10^. Since wild-type parkin interacts with the C-terminal tail of GluK2 and is able to ubiquitinate GluK2^9^, GluK2 accumulation in parkinQ311X-DA neurons may result from decreased proteasome-mediated degradation. We speculate that GluK2 ubiquitination by parkin might also modulate KAR endo–exocytosis, surface localization, or lateral diffusion between synaptic and extrasynaptic membrane.

KAR function in DA neurons is still unexplored. Spontaneous firing-frequency sensitivity to UBP310 is increased in parkinQ311X-DA neurons. These data suggest that KARs can participate in the regulation of DA neuron firing frequency. The spontaneous activity pattern of adult SNc DA neurons in vivo results from interactions between intrinsic pacemaker mechanisms and synaptic inputs. Since only the pacemaker-like pattern can be recorded in vitro^27–29^, our data suggest that the expression of parkin mutants in SNc DA neurons affects their pacemaker activity. Pacemaker activity is associated with DA release and intrinsically driven by voltage-gated Cav1.3 calcium channels. We speculate that KAR channel gating may facilitate membrane depolarization and activation of voltage-gated Cav1.3 calcium channels. A previous study showed an increase in the spontaneous firing frequency of DA neurons in a PD model generated by overexpressing mutant α-synuclein^30^. This suggests that the increase in the spontaneous firing of DA neurons may be a common feature of PD forms and that UBP310 may revert this dysfunction.

Because of their large fractional Ca^2+^ influx during action potential and their enormously large axonal tree, SNc DA neurons have a constitutive higher metabolic demand than other neurons^31^. By increasing KAR-mediated glutamatergic transmission, *PARK2* mutations can lead to early dysregulation of pacemaker activity and Ca^2+^ influx in SNc DA neurons, thus increasing their energy costs and susceptibility to oxidative damage, Ca^2+^ overload, and excitotoxicity. We observed KAR accumulation and dysregulation of the spontaneous firing frequency in DA neurons of parkinQ311X at 1 month of age. These dysfunctional features preceded DA neuron loss that became evident at 6 months of age. If these data are confirmed in other parkin models, they configure KAR accumulation and excitability dysregulation as early and potentially reversible toxic events that precede DA neuron death, which is conversely a nonreversible process. As early and reversible processes, in principle, they can be pharmacologically targeted to prevent DA neuron death.

Consistent with this hypothesis, we found that treatment with KAR antagonist UBP310 for 3 and 4.5 months in vivo prevents DA neuron degeneration caused by parkinQ311X expression.

This finding is in line with a recent study that showed that UBP310 can rescue DA neuron loss induced by the parkinsonian toxin 1-methyl-4-phenyl-1,2,3,6-tetrahydropyridine in mice^32^, further confirming the disease-modifying potential of KAR antagonism in PD.

UBP310 has been reported to be a potent antagonist at KARs^22–24,33,34^. UBP310 is a 4000-fold more potent antagonist at KAR than AMPAR and is ineffective at NMDAR and metabotropic glutamate receptors^24^. UBP310 blocked postsynaptic KAR at hippocampal mossy fiber CA3 synapses while sparing AMPAR and NMDAR^22^. UBP310 prevented epileptic activity in WT mice but was ineffective in mice knockout for the GluK2 subunit of KAR^23^. Hence, several lines of evidence show that UBP310 is a specific KAR antagonist.

We found that KAR antagonism in parkinQ311X mice reduced the GluK2 levels in brain tissues and in DA neurons; this finding confirms the effects of UBP310 on KARs. In WT mice, UBP310 decreased GluK2 levels in 4 h, whereas, in parkinQ311X mice, the decrease in GluK2 levels occurred 8 h after the treatment. Considering that parkin ubiquitinates GluK2 and this post-transductional modification may mediate GluK2 turnover, we can hypothesize that mutant parkin expression may slow down GluK2 turnover. As previously suggested, it is still possible that the neuroprotective effect of UBP310 in the midbrain may result from its action on KARs formed by the combination of different subunits^32^.

In conclusion, this study provides novel evidence of a causal role of KARs in the DA neuron dysfunction and loss occurring in a mouse model of human parkin-induced toxicity, and supports KAR as a novel target in neuroprotective therapy of nigral DA neurons. The favorable safety profile of chronic UBP310 in mice paves the way to further analyses, which might extend to other preclinical models of ARJP as well as idiopathic PD.

## Supplementary information

Figure S1

Figure S2

Figure S3

Figure S4

Figure S5

## References

[1] Kitada T (1998). Mutations in the parkin gene cause autosomal recessive juvenile parkinsonism. Nature.

[2] Zhang C-W, Hang L, Yao T-P, Lim K-L (2015). Parkin regulation and neurodegenerative disorders. Front. Aging Neurosci..

[3] Scarffe LA, Stevens DA, Dawson VL, Dawson TM (2014). Parkin and PINK1: much more than mitophagy. Trends Neurosci..

[4] Charan RA, LaVoie MJ (2015). Pathologic and therapeutic implications for the cell biology of parkin. Mol. Cell. Neurosci..

[5] Sassone J (2017). The synaptic function of parkin. Brain.

[6] Kubo S (2001). Parkin is associated with cellular vesicles. J. Neurochem..

[7] Fallon L (2002). Parkin and CASK / LIN-2 associate via a PDZ-mediated interaction and are co-localized in lipid rafts and postsynaptic densities in brain. J. Biol. Chem..

[8] Joch M (2007). Parkin-mediated Monoubiquitination of the PDZ Protein PICK1 regulates the activity of acid-sensing ion channels. Mol. Biol. Cell..

[9] Maraschi A (2014). Parkin regulates kainate receptors by interacting with the GluK2 subunit. Nat. Commun..

[10] Cremer JN (2015). Changes in the expression of neurotransmitter receptors in Parkin and DJ-1 knockout mice—a quantitative multireceptor study. Neuroscience.

[11] Porter RHP, Eastwood SL, Harrison PJ (1997). Distribution of kainate receptor subunit mRNAs in human hippocampus, neocortex and cerebellum, and bilateral reduction of hippocampal GluR6 and KA2 transcripts in schizophrenia. Brain Res..

[12] Mueller HT, Haroutunian V, Davis KL, Meador-Woodruff JH (2004). Expression of the ionotropic glutamate receptor subunits and NMDA receptor-associated intracellular proteins in the substantia nigra in schizophrenia. Mol. Brain Res..

[13] Lerma J, Marques JM (2013). Kainate receptors in health and disease. Neuron.

[14] Contractor A, Mulle C, Swanson GT (2011). Kainate receptors coming of age: milestones of two decades of research. Trends Neurosci..

[15] Hattori N (1998). Point mutations (Thr240Arg and Ala311Stop) in the Parkin gene. Biochem. Biophys. Res. Commun..

[16] Lu X-H (2009). Bacterial artificial chromosome transgenic mice expressing a truncated mutant parkin exhibit age-dependent hypokinetic motor deficits, dopaminergic neuron degeneration, and accumulation of proteinase K-resistant alpha-synuclein. J. Neurosci..

[17] Schneider CA, Rasband WS, Eliceiri KW (2012). NIH Image to ImageJ: 25 years of image analysis. Nat. Methods.

[18] Novello S (2018). G2019S LRRK2 mutation facilitates α-synuclein neuropathology in aged mice. Neurobiol. Dis..

[19] Paxinos, G. & Franklin, K. B. J. *Paxinos and Franklin’s the Mouse Brain in Stereotaxic Coordinates*. (Academic Press, 2001).

[20] Arcuri L (2016). Genetic and pharmacological evidence that endogenous nociceptin/orphanin FQ contributes to dopamine cell loss in Parkinson’s disease. Neurobiol. Dis..

[21] Larsen JO, Gundersen HJG, Nielsen J (1998). Global spatial sampling with isotropic virtual planes: estimators of length density and total length in thick, arbitrarily orientated sections. J. Microsc..

[22] Pinheiro PS (2013). Selective block of postsynaptic kainate receptors reveals their function at hippocampal mossy fiber synapses. Cereb. Cortex..

[23] Peret A (2014). Contribution of aberrant GluK2-containing kainate receptors to chronic seizures in temporal lobe epilepsy. Cell Rep..

[24] Dolman NP (2007). Synthesis and pharmacological characterization of N3-substituted willardiine derivatives: Role of the substituent at the 5-position of the uracil ring in the development of highly potent and selective GLUK5 kainate receptor antagonists. J. Med. Chem..

[25] Fricker M, Tolkovsky AM, Borutaite V, Coleman M, Brown GC (2018). Neuronal cell death. Physiol. Rev..

[26] Jaskolski F, Coussen F, Mulle C (2005). Subcellular localization and trafficking of kainate receptors. Trends Pharmacol. Sci..

[27] Richards CD, Shiroyama T, Kitai ST (1997). Electrophysiological and immunocytochemical characterization of GABA and dopamine neurons in the substantia nigra of the rat. Neuroscience.

[28] Morikawa H, Paladini CA (2011). Dynamic regulation of midbrain dopamine neuron activity: intrinsic, synaptic, and plasticity mechanisms. Neuroscience.

[29] Roeper J (2013). Dissecting the diversity of midbrain dopamine neurons. Trends Neurosci..

[30] Subramaniam M (2014). Mutant-synuclein enhances firing frequencies in dopamine substantia nigra neurons by oxidative impairment of A-type potassium channels. J. Neurosci..

[31] Bolam JP, Pissadaki EK (2012). Living on the edge with too many mouths to feed: why dopamine neurons die. Mov. Disord..

[32] Stayte S (2020). The kainate receptor antagonist UBP310 but not single deletion of GluK1, GluK2, or GluK3 subunits, inhibits MPTP-induced degeneration in the mouse midbrain. Exp. Neurol..

[33] Atlason PT (2010). Mapping the ligand binding sites of kainate receptors: Molecular determinants of subunit-selective binding of the antagonist [3H]UBP310. Mol. Pharmacol..

[34] Perrais D, Pinheiro PS, Jane DE, Mulle C (2009). Antagonism of recombinant and native GluK3-containing kainate receptors. Neuropharmacology.

