# Supplementary figures and images for: Pharmacological antagonism of kainate receptor rescues dysfunction and loss of dopamine neurons in a mouse model of human parkin-induced toxicity
Source: Cell Death Dis. 2020 Nov 10;11(11):963. doi: 10.1038/s41419-020-03172-8 (PMC7656261; doi:10.1038/s41419-020-03172-8)

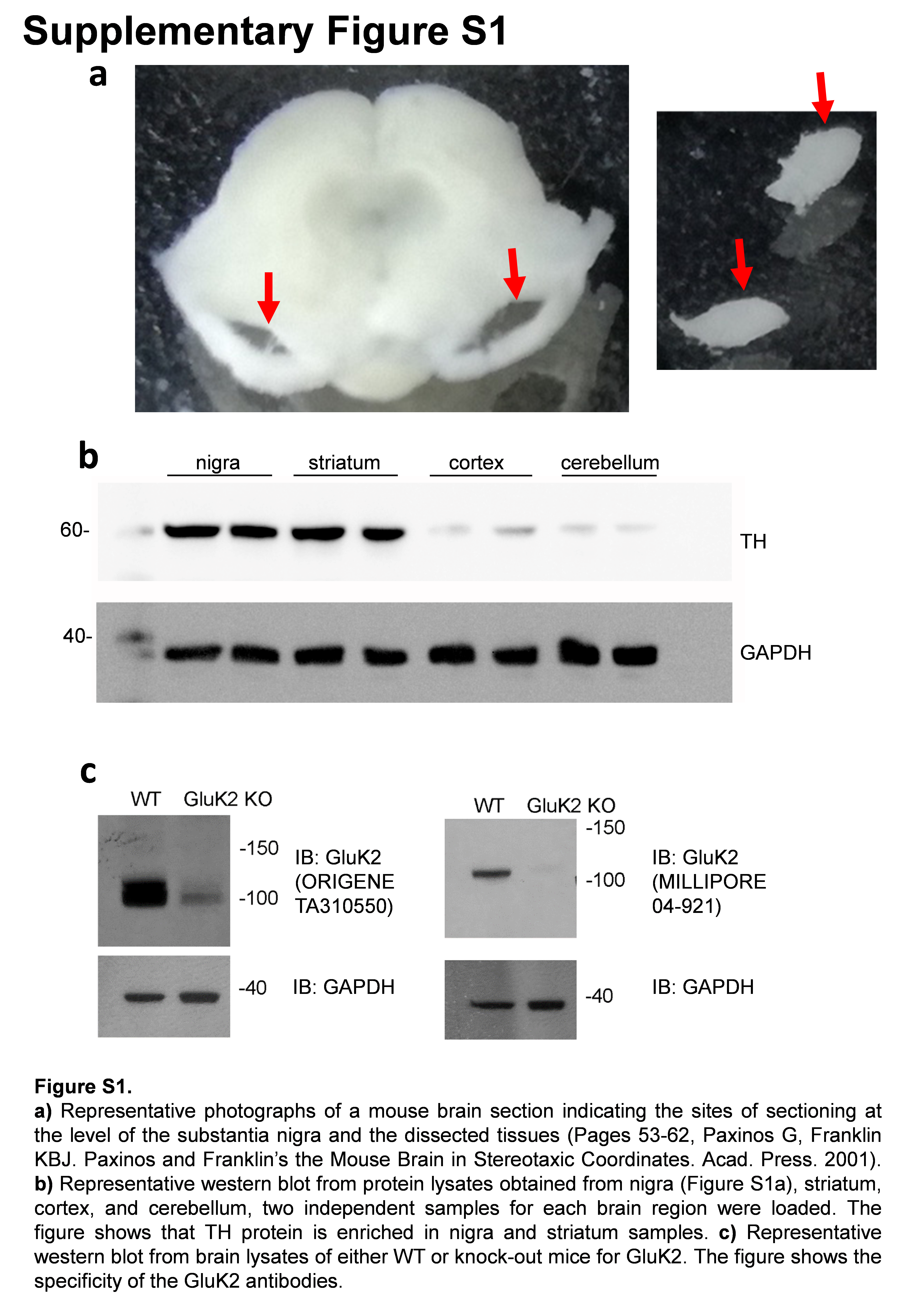

Supplement: Supplementary file 1 — Figure S1 [file 41419_2020_3172_MOESM1_ESM.tif]

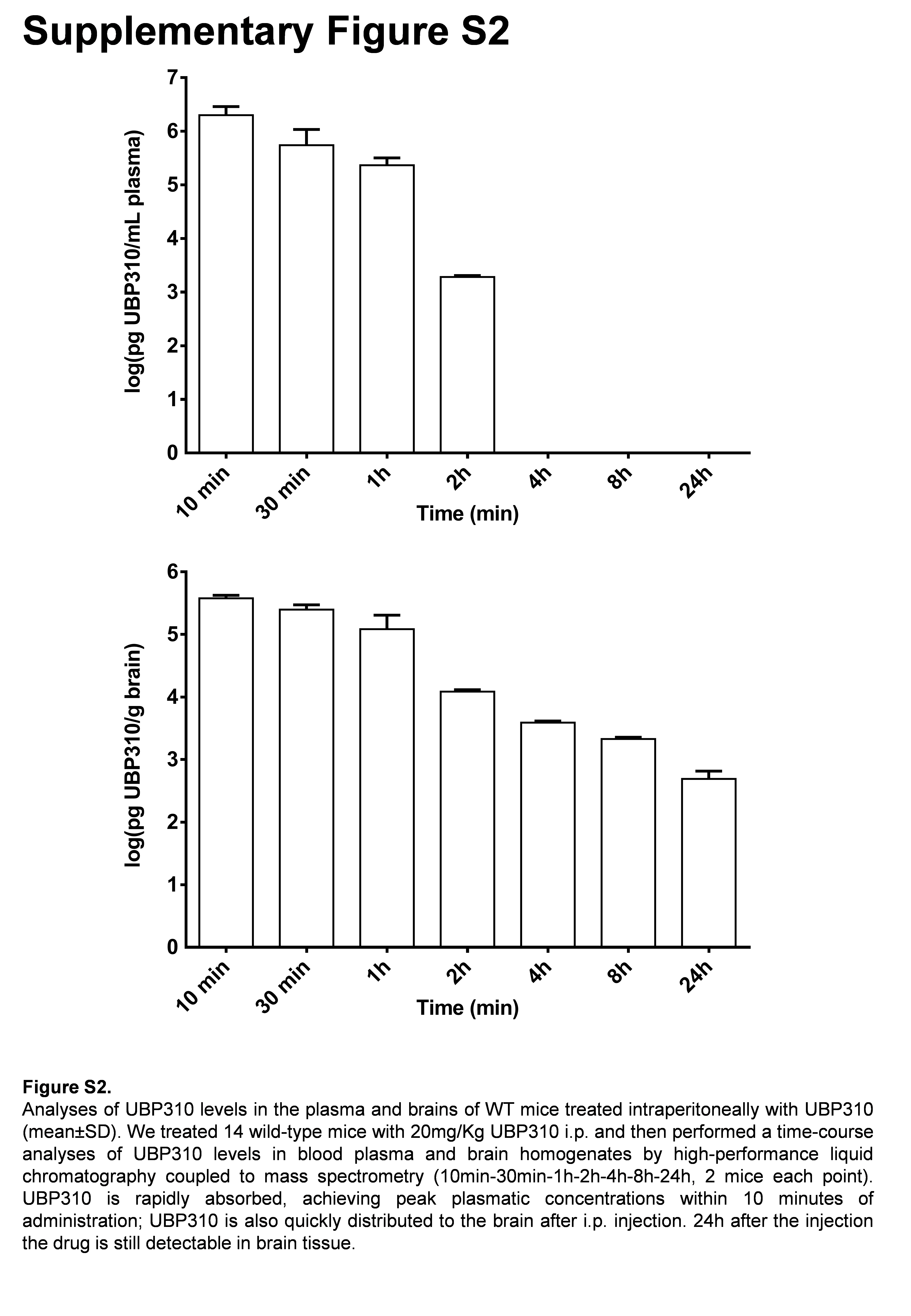

Supplement: Supplementary file 2 — Figure S2 [file 41419_2020_3172_MOESM2_ESM.tif]

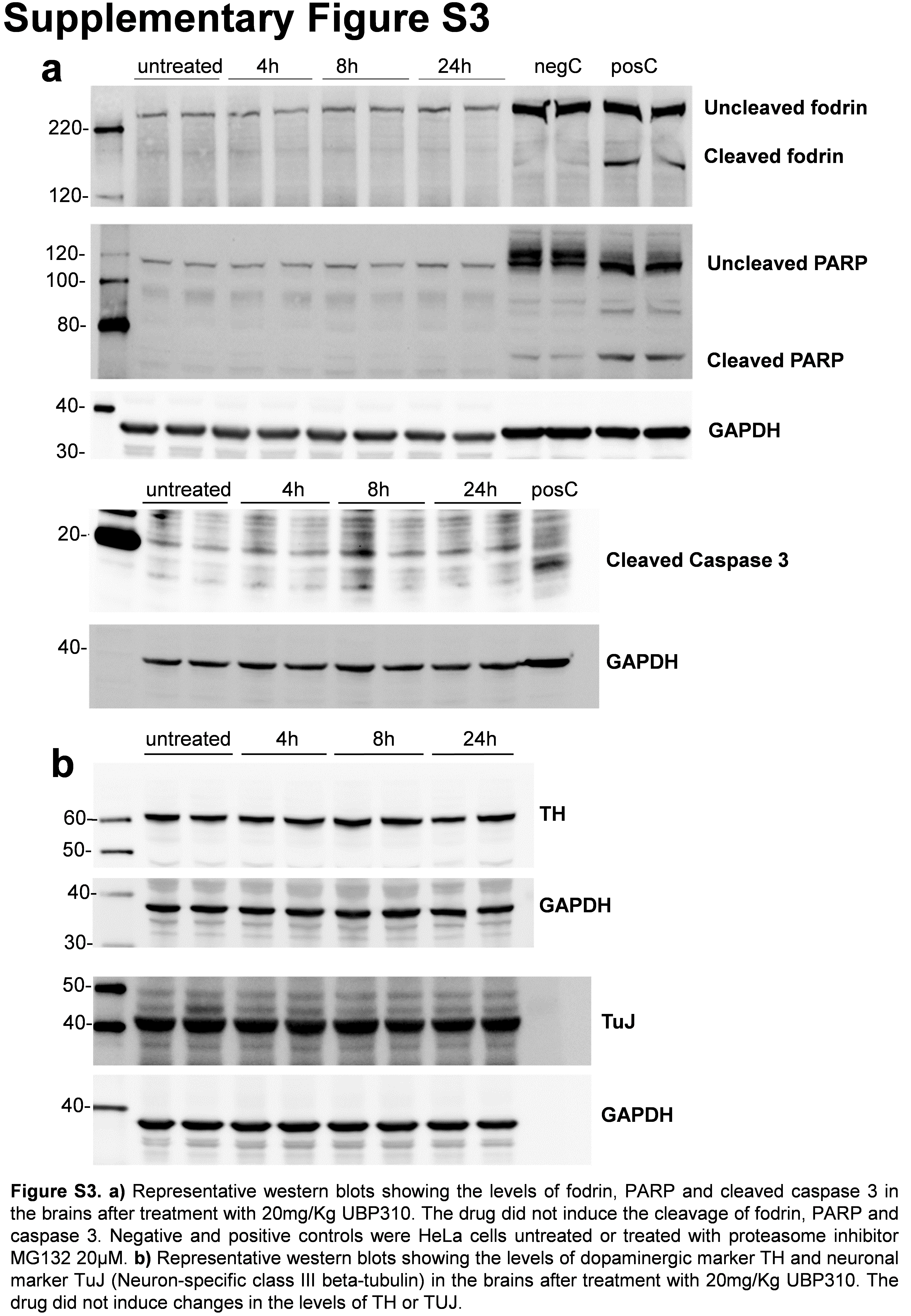

Supplement: Supplementary file 3 — Figure S3 [file 41419_2020_3172_MOESM3_ESM.tif]

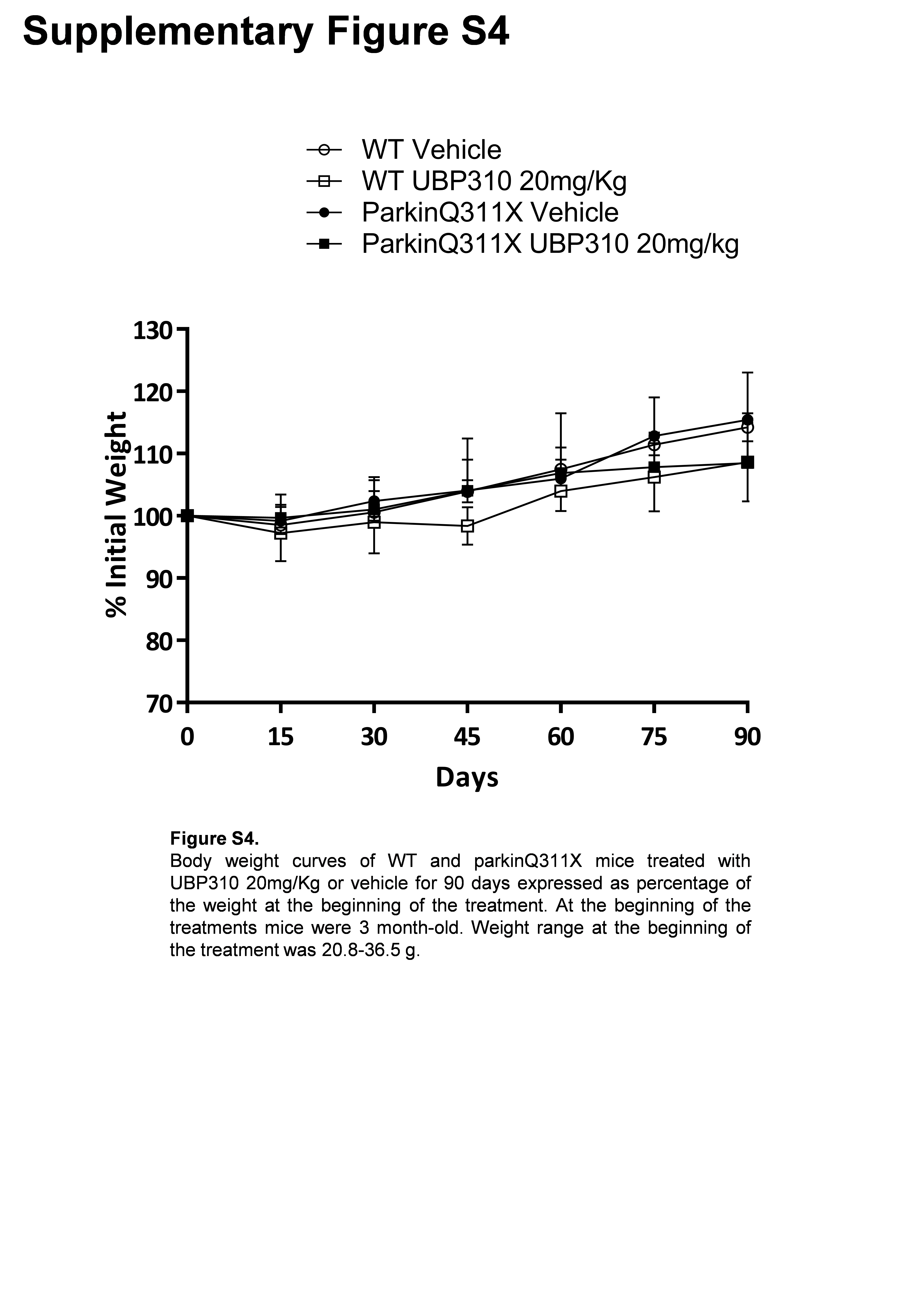

Supplement: Supplementary file 4 — Figure S4 [file 41419_2020_3172_MOESM4_ESM.tif]

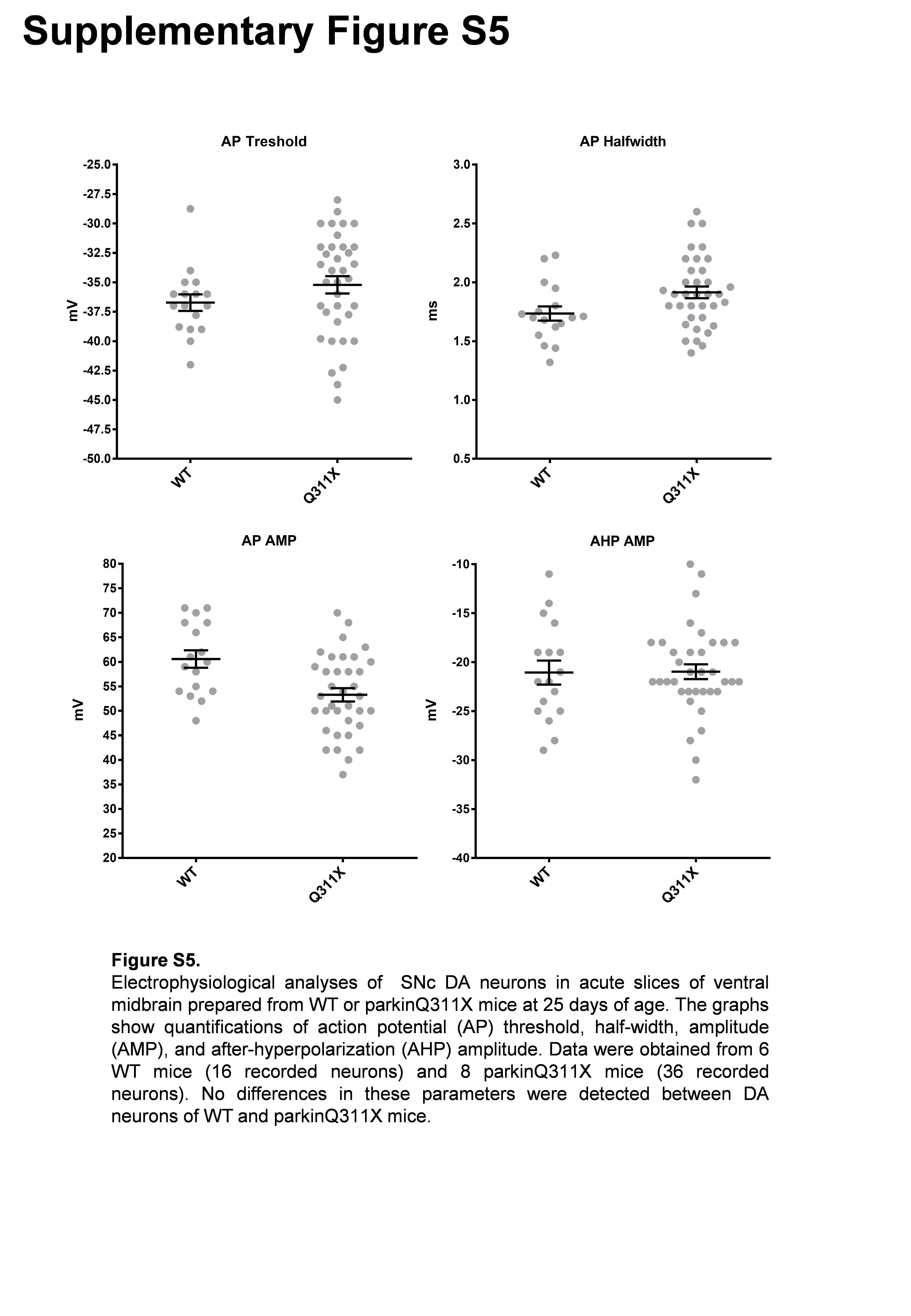

Supplement: Supplementary file 5 — Figure S5 [file 41419_2020_3172_MOESM5_ESM.tif]
